# Internet-Based Behavioral Activation for Depression: Systematic Review and Meta-Analysis

**DOI:** 10.2196/41643

**Published:** 2023-05-25

**Authors:** Carolin Sophie Alber, Lena Violetta Krämer, Sophia Marie Rosar, Claudia Mueller-Weinitschke

**Affiliations:** 1 Department of Rehabilitation Psychology and Psychotherapy Institute of Psychology University of Freiburg Freiburg Germany

**Keywords:** behavioral activation, depression, systematic review, meta-analysis, internet- and mobile-based intervention

## Abstract

**Background:**

Behavioral activation is an effective treatment for reducing depression. As depressive disorders affect many people worldwide, internet-based behavioral activation (iBA) could provide enhanced treatment access.

**Objective:**

This study aimed to investigate whether iBA is effective in reducing depressive symptoms and to assess the impact on secondary outcomes.

**Methods:**

We systematically searched MEDLINE, PsycINFO, PSYNDEX, and CENTRAL up to December 2021 for eligible randomized controlled trials. In addition, a reference search was conducted. Title and abstract screening, as well as a full-text screening, was conducted by 2 independent reviewers. Randomized controlled trials that investigated the effectiveness of iBA for depression as a treatment or main component were included. Randomized controlled trials had to report depressive symptoms, with a quantitative outcome measure and assess an adult population with depressive symptoms above cutoff. Two independent reviewers performed the data extraction and risk of bias assessment. Data were pooled in random-effects meta-analyses. The primary outcome was self-reported depressive symptoms posttreatment. This systematic review and meta-analysis followed the Preferred Reporting Items for Systematic Reviews and Meta-Analyses (PRISMA) reporting guidelines.

**Results:**

A total of 12 randomized controlled trials, with 3274 participants (88% female, 43.61 years) were included. iBA was more effective in reducing depressive symptom severity posttreatment than inactive control groups (standardized mean difference −0.49; 95% CI −0.63 to −0.34; *P*<.001). The overall level of heterogeneity was moderate to substantial (*I*^2^=53%). No significant effect of iBA on depressive symptoms could be found at 6-month follow-up. Participants assigned to iBA also experienced a significant reduction of anxiety and a significant increase in quality of life and activation compared to the inactive control groups. The results remained robust in multiple sensitivity analyses. The risk of bias assessment revealed at least some concerns for all studies, and there was evidence of slight publication bias.

**Conclusions:**

This systematic review and meta-analysis implies that iBA is effective in reducing depressive symptoms. It represents a promising treatment option, providing treatment access where no treatment is available yet.

**Trial Registration:**

International Prospective Register of Systematic Reviews CRD42021236822; https://www.crd.york.ac.uk/prospero/display_record.php?RecordID=236822

## Introduction

Depression is a worldwide health problem reducing the quality of life and increasing the relative risk for mortality [1-3]. Still, an insufficient number of treatment options for depression exists, increasing the risk of chronification [4]. New treatment options, which can provide enhanced treatment access, are urgently needed [5].

Behavioral activation (BA) is an effective treatment for reducing depressive symptoms [6]. Originally developed by Lewinsohn in 1974, the BA framework suggests that depression evolves due to low activity levels and an insufficient level of response-contingent reinforcement [7]. Behavioral treatment elements (eg, activity monitoring, activity scheduling, and values assessment) aim to increase a patient’s daily activities and access to positive reinforcement [8]. Various studies and meta-analyses show that BA is effective as a stand-alone treatment in reducing depression [9-11] and comparably effective to cognitive behavioral therapy (CBT) [9,12-14]. BA holds several advantages: It contains fewer treatment elements than CBT, making it parsimonious and easily comprehensible to patients [15,16]. It is simple to deliver and needs little training and experience from therapists [13,15]. BA can potentially reduce the need for costly professional training, increase access to psychological therapies, and reduce waiting times [6]. It might therefore be particularly suitable for implementation via the internet [17].

There exist several studies implementing internet-based BA (iBA) interventions [18-22], and few reviews on iBA [17,23]. The option to conduct a comprehensive meta-analysis on iBA was previously limited due to the small number of primary studies on iBA. The previous reviews [17,23] had to be built on heterogeneous populations, delivery modes, and intervention rationales, resulting in high clinical heterogeneity. Despite these constraints, a meta-analysis showed promising results in favor of iBA (standardized mean difference [SMD] −0.67; 95% CI −0.96 to −0.37; *P*<.001) [17]. Yet, the high clinical heterogeneity made it difficult to draw valid conclusions on the effectiveness of iBA. Publication bias could not be assessed due to the limited number of included studies.

In our meta-analysis, we want to provide a full picture of iBA effectiveness, including the most recent developments in iBA research. To address the methodological and practical limitations of the former meta-analysis [17] and especially reduce clinical heterogeneity, we specify and narrow the inclusion criteria by reducing the variety of eligible study populations and increasing the similarity of interventions regarding their content elements and delivery mode. The following main research questions are addressed: (1) is iBA effective in treating depressive symptoms compared to inactive control groups (CGs)? (2) How does iBA compare to other treatments concerning the effectiveness for the treatment of depressive symptoms? (3) Does iBA have an effect on secondary outcomes?

## Methods

### Preregistration and Reporting

This systematic review and meta-analysis was preregistered in the International Prospective Register of Systematic Reviews (CRD42021236822). The reporting follows the Preferred Reporting Items for Systematic Reviews and Meta-Analyses (PRISMA) guidelines [24] (see Multimedia Appendix 1). Amendments and specifications to the PROSPERO registration can be found in Multimedia Appendix 2.

### Search Strategy and Selection Criteria

MEDLINE, PsycINFO, and PSYNDEX (via EBSCO), as well as CENTRAL, were searched for eligible records from database inception until December 13, 2021.

Suitable for inclusion were the following: (1) randomized controlled trials (RCTs), (2) written in English or German, that (3) assessed an adult sample (aged 18 years or older) of depressed individuals (symptoms above predefined cutoff score on depression measures), that (4) evaluated the effectiveness of an internet-based intervention, with (5) BA as an exclusive treatment or main component, and (6) reported depressive symptom severity as a quantitative outcome measure. There were no restrictions on CGs, further sociodemographic characteristics of participants, or publication date. Studies containing videoconferencing or telephone-based therapy were excluded as they resemble face-to-face therapy.

The search string (Multimedia Appendix 3) included a combination of terms concerning digital health, BA, depression, and RCTs. For each category, we derived search terms based on search terms of former meta-analyses [9,11,17,25] and theoretical considerations (CSA, CM-W, and LVK). Different from previous meta-analyses [9,17], the search term excluded near-operators and the umbrella term “behavior therapy,” as we classified these terms as too unspecific for the distinct field of BA. In the last step, this search string was adapted for each database’s requirements. The quality of the search string was evaluated using a predefined validation set including all relevant studies from previous meta-analyses (Multimedia Appendix 4) [9,17]. All studies of our validation set were detected. Reference lists of included studies were searched for additional eligible records.

Title and abstract screening, as well as full-text screening, was fully conducted by 2 independent reviewers (CSA and CM-W). Reasons for the exclusion of studies were recorded throughout the process. Discrepancies were resolved in discussion with a third reviewer (LVK). The selection process was based on a prior developed inclusion criteria checklist (Multimedia Appendix 5).

### Data Extraction

Data were extracted independently by 2 reviewers (CSA and SMR) using a previously established data extraction sheet. The following data were extracted: data describing the (1) publication of the study, (2) population, (3) interventions, (4) comparators, (5) study design, (6) outcomes, and (7) study characteristics (Multimedia Appendix 6).

Data were extracted for the main outcome of depressive symptoms as well as anxiety, quality of life, and activation as secondary outcomes. If necessary data were not reported, we contacted the corresponding authors via email. All requested data were provided. If multiple measures were used, data extraction was prioritized as follows: (1) validated questionnaires, (2) clinical ratings, and (3) single-item analysis. Wherever possible, intention-to-treat (ITT) data were used. ITT data were defined as analysis based on assignment to the intervention, meaning all randomized participants were included in the analysis.

### Data Analysis

We conducted a random-effects meta-analysis for the main outcome of depressive symptoms (main analysis) against inactive CGs at posttreatment. In addition to the main analysis on inactive CGs, we conducted random-effects meta-analyses for the outcome of depressive symptoms against active CGs (cognitive interventions and mindfulness). Subgroup and sensitivity analyses were performed for the main analysis (inactive CGs) only.

Subgroup analyses were preplanned, investigating differences regarding (1) the mode of delivery of iBA via browser-based digital intervention or smartphone app and (2) the amount of guidance (unguided intervention/minimal guidance/guided intervention; for the classification see Multimedia Appendix 7) within iBA interventions.

Sensitivity analyses served to evaluate the robustness of the effect retrieved from the main analysis. We repeated the main analysis but used (1) the alternative outcome measure for depressive symptoms if multiple instruments were used in the respective primary study. Additionally, we conducted sensitivity analyses with respective subsamples of studies that (2) investigated depressive symptoms, excluding postnatal depression, (3) reported ITT data, (4) did not include any additional module content other than BA, (5) reported depressive symptoms above this meta-analysis’ predefined cutoff, excluding studies which reported only mild depressive symptoms at baseline, (6) were of acceptable methodological quality, excluding interventions that were at high risk of bias and, (7) reported data for adult samples only, excluding the study that reported data for participants at the age of 16 years or older [18].

We conducted a post hoc meta-analysis for the main outcome of depressive symptoms at the 6-month follow-up to investigate the stability of effects, as this was the longest follow-up time point for which results from more than 2 data sets were available. The effect of iBA on depressive symptoms at posttreatment was also assessed compared to cognitive interventions (CBT/cognitive therapy) and mindfulness, as more than 1 study included these CGs. Secondary outcome analyses were conducted comparing iBA against inactive CGs at posttreatment if more than 2 comparisons were available.

Data analysis was performed using Review Manager (RevMan 5.4) [26] for Windows provided by the Cochrane Collaboration. We estimated SMDs using Hedges *g* and 95% CIs. Inverse variance was used to weight studies. Two-sided *P*<.05 indicated statistical significance. Statistical heterogeneity of studies was judged using *I*^2^ statistics and interpreted according to the GRADE handbook. An *I*^2^ score of less than 40% indicates low, 30%-60% moderate, 50%-90% substantial, and 75%-100% considerable heterogeneity [27]. Publication bias was assessed visually via a funnel plot for the primary outcome of depressive symptoms at posttreatment.

### Risk of Bias

Two reviewers (CSA and SMR) independently rated the risk of bias of all included studies using the Cochrane Collaboration’s Risk of Bias tool (RoB 2 [28]). Disagreements were resolved by discussion with a third reviewer (CM-W).

## Results

### Study Selection

**Figure 1:**
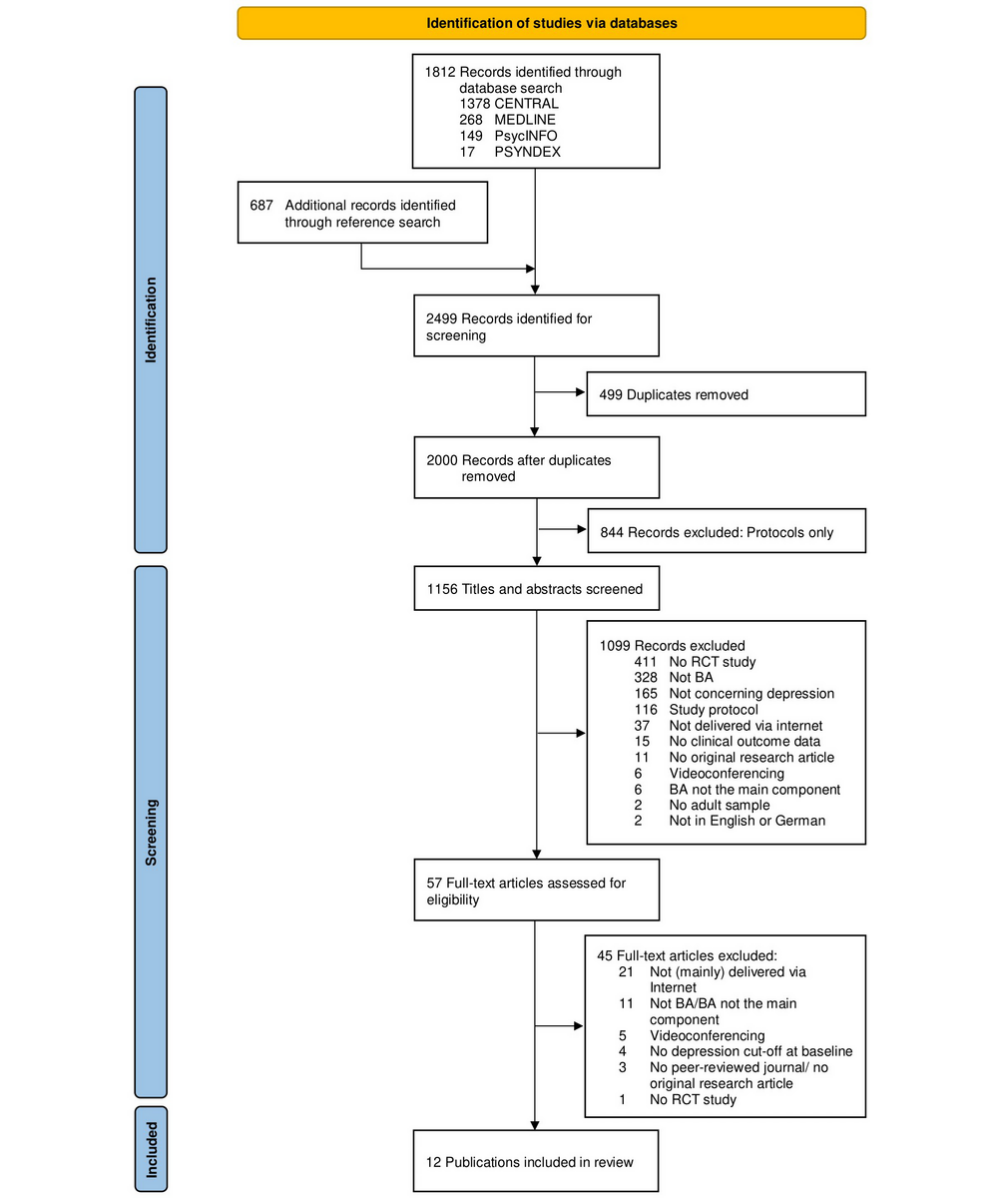
PRISMA (Preferred Reporting Items for Systematic Reviews and Meta-Analyses) flowchart. BA: behavioral activation; RCT: randomized controlled trial.

We identified 2499 records for screening, of which 12 publications met inclusion criteria. As 1 publication included 2 separate studies, a total of 13 studies [18-22,29-35] could be included in this systematic review, and 12 studies could be included in the quantitative meta-analysis [18-22,29,30,32-35] with 3274 baseline participants (see flowchart in Figure 1).

### Study Characteristics

Study characteristics for the included studies are provided in Table 1. Included studies were published between 2013 and 2021. All studies investigated adult samples. One study had an age cutoff of 16 years, but the sample consisted of 97.4% participants aged 18 years or older (mean 24.48, SD 5.07 years) [18]. The mean age of all included participants in the meta-analysis was 43.61 (SD 9.91) years. The proportion of female participants ranged from 67% to 100%. Two studies included exclusively women with postpartum depression [33,34]; all other studies investigated general depression. The majority of studies were conducted in Western Europe [19,21,30-34] and the United States [20,29,35]. One study each was conducted in Indonesia [18], Brazil, and Peru [22]. Most studies compared an iBA intervention with an inactive CG: either treatment as usual [18,20-22,29,33,34] or waitlist control group [19,30,32,35] as defined by the authors. Some studies had additional active CGs (CBT [20,29]; cognitive therapy [35]; and mindfulness [21]), and 1 study compared with an active CG only (mindfulness [31]) and was therefore not included in the main analysis. There was 1 study [32] that investigated 2 BA interventions based either on the BA model of Martell or Lewinsohn. As this study presented only 1 CG, we included only the BA intervention based on Martell model of BA to prevent repeating the standard error of this CG. iBA interventions in primary studies were either transmitted as browser-based digital interventions [18,19,21,30,32-34] or via smartphone app [20,22,29,31,35]. The length of iBA interventions ranged from 2 to 15 weeks, from 6 to 18 modules, and from 10 minutes to 1 hour for each module. Posttreatment assessment varied from 2 to 17 weeks after baseline, depending on intervention length. There were 3 unguided interventions [20,21,29] and 4 interventions providing minimal guidance [19,22,30], consisting of technical and administrative support or motivational reminders. The remaining 6 interventions were guided [18,31-35] and used limited therapist support via telephone or messaging service. While most studies implemented pure iBA interventions, there were 4 studies that included minimal additional modules: acceptance and mindfulness of thoughts [19], problem-solving [30], and rumination [33,34] (details on intervention description are displayed in Multimedia Appendix 8).

All studies used validated questionnaires to measure depressive symptoms as well as the respective secondary outcomes if these were available (Table 1). An additional clinical rating for depression was used in 1 study [19]. A follow-up measure at 6 months after baseline was offered by 2 studies [18,22]. Of the 12 studies included in the main analysis, the reported data of 10 studies was based on assigned randomization [18,19,21,22,30,32-35], of which 6 studies provided estimations for missing data (eg, via multiple imputations) and were classified as ITT [18,19,31,32,34,35].

**Table 1:** Study characteristics.

Source	Country	Population (cutoff for inclusion)	Baseline (female), n (%)	Age at baseline (years), mean (SD)	Intervention	Control groups	Measure time points		Outcomes (measures)	ITT-data available^a,b^	Dropout rate at posttreatment (%)	
							Posttreatment in weeks	Follow-up in months			IG^c^	CG^d^
Araya et al, 2021 [22]	Brazil	Adults with depressive symptoms (PHQ-9 ≥10),^e^ hypertension and diabetes	880 (86.48)	56.02 (11.59)^f^	Smartphone-based BA^g^ app (CONEMO app)	Enhanced usual care	12	6	Depressive symptoms (PHQ-9), quality of life (EQ-5D-3L),^h^ activation (BADS-SF)^i^	No	49 (11)	41 (9)
Araya et al, 2021 [22]	Peru	Adults with depressive symptoms (PHQ-9≥10), hypertension and diabetes	432 (81.48)	59.75 (11.22)^f^	Smartphone-based BA app (CONEMO app)	Enhanced usual care	12	6	Depressive symptoms (PHQ-9), quality of life (EQ-5D-3L), activation (BADS-SF)	No	12 (6)	10 (5)
Arjadi et al, 2018 [18]	Indonesia	Individuals with MDD^j^ or PDD^k^ diagnosis (PHQ-9≥10)	313 (81)	24.48 (5.07)	Browser-based digital BA intervention (Guided Act and Feel Indonesia)	TAU^l^	10	3, 6	Depressive symptoms (PHQ-9), quality of life (WHO-QOL-BREF)^m^	Yes	39 (25)	9 (6)
Carlbring et al, 2013 [19]	Sweden	Adults with MDD diagnosis (MADRS 15-30)^n^	80 (82.5)	44.4 (13.5)	Browser-based digital BA intervention (Depressionshjälpen), minimal modules of ACT^o^	WLC^p^	8	3^q^	Depressive symptoms (BDI-II),^r^ anxiety (BAI),^s^ quality of life (QOLI)^t^	Yes	0 (0)	2 (5)
Dahne et al, 2019 (Moodivate) [20]	United States	Adults with depressive symptoms (PHQ-8^u^ ≥ 10)	52 (84.6)	43.79 (13.27)	Smartphone-based BA app (Moodivate)	CBT^v^ and TAU	8	None	Depressive symptoms (BDI-II)	No	5 (21)	5 (26); 1 (11)
Dahne et al, 2019 (Aptívate) [29]	United States	Adults with depressive symptoms (PHQ-8 ≥10)	42 (66.7)	36.05 (11.44)	Smartphone-based BA app (Aptívate)	CBT and TAU	8	None	Depressive symptoms (BDI-II)	No	3 (14)	1 (11); 5 (45)
Jelinek et al, 2020 [21]	Germany	Adults with depressive symptoms (PHQ-9 ≥4)	104 (76.9)	46.21 (10.24)	Browser-based digital BA intervention	Mindfulness and TAU	2	1	Depressive symptoms (PHQ-9), quality of life (WHOQOL-BREF)	No	8 (22)	5 (16); 3 (9)
Lambert et al, 2018 [30]	United Kingdom	Adults with depressive symptoms (PHQ-8 ≥ 10)	62 (84)	38.1 (12.3)	Browser-based digital BA intervention (eMotion)	WLC	8	None	Depressive symptoms (PHQ-8)	No	7 (22)	5 (17)
Ly et al, 2014 [31]	Sweden	Adults with MDD diagnosis (PHQ-9 ≥5)	81 (70)	36.1 (10.8)	Smartphone-based BA app	Mindfulness	8	6	Depressive symptoms (PHQ-9), anxiety (BAI), quality of life (QOLI)	Yes	4 (10)	5 (12)
Nyström et al, 2017 [32]	Sweden	Adults with MDD diagnosis (MADRS 15-35)	286 (76)	42.0 (13.5)	Browser-based digital BA intervention (Lewinsohn), browser-based digital BA intervention (Martell)^w^	Physical activity without rational, physical activity with rational, WLC	12	None	Depressive symptoms (PHQ-9), anxiety (GAD-7)^x^	Yes	4 (6)	9 (18); 12 (20); 7 (11); 5 (09)
O'Mahen et al, 2013 [34]	United Kingdom	Women with depressive symptoms (EPDS>12)^y^	910 (100)	32.2 (5.2)	Browser-based digital BA intervention (postnatal iBA)	TAU	15	None	Depressive symptoms (EPDS)	Yes	281 (60.8)	286 (63.9)
O'Mahen et al, 2014 [33]	United Kingdom	Women with MDD diagnosis (EPDS > 12)	83 (100)	NA^z^	Browser-based digital BA intervention (NetmumsHWD)	TAU	17	10	Depressive symptoms (EPDS), anxiety (GAD-7)	No	3 (3.6)	8 (9.6)
Stiles-Shields et al, 2019 [35]	United States	Adults with depressive symptoms (PHQ-9 ≥ 10)	30 (76)	37.6 (13.2)	Smartphone-based BA app (Boost Me)	CT^aa^ and WLC	6	2.5	Depressive symptoms (PHQ-9)	Yes	0 (0)	3 (10); 0 (0)

^a^ITT: intention-to-treat.

^b^Means and SDs are for complete baseline N available.

^c^IG: intervention group.

^d^CG: control group.

^e^PHQ-9: Patient Health Questionnaire-9.

^f^Means and SDs for complete participant population were calculated from group-based means and SDs.

^g^BA: behavioral activation.

^h^EQ-5D-3L: Three-Level Version of EuroQol Five-Dimensional Questionnaire.

^i^BADS-SF: Behavioral Activation for Depression Scale – Short Form.

^j^MDD: major depressive disorder.

^k^PDD: persistent depressive disorder.

^l^TAU: treatment as usual.

^m^WHO-QOL-BREF: WHO-Quality of Live Questionnaire.

^n^MADRS: Montgomery-Åsberg Depression Rating Scale.

^o^ACT: acceptance and commitment therapy.

^p^WLC: waitlist control group.

^q^Only the intervention group completed the follow-up.

^r^BDI-II: Beck Depression Inventory.

^s^BAI: Beck Anxiety Inventory.

^t^QOLI: Quality of Life Inventory.

^u^PHQ-8: Patient Health Questionnaire-8.

^v^CBT: cognitive behavioral therapy.

^w^Included in main analysis.

^x^GAD-7: Generalized Anxiety Disorder 7-item Scale.

^y^EPDS: Edinburgh Postnatal Depression Scale.

^z^NA: not available.

^aa^CT: cognitive therapy.

### Risk of Bias

**Figure 2:**
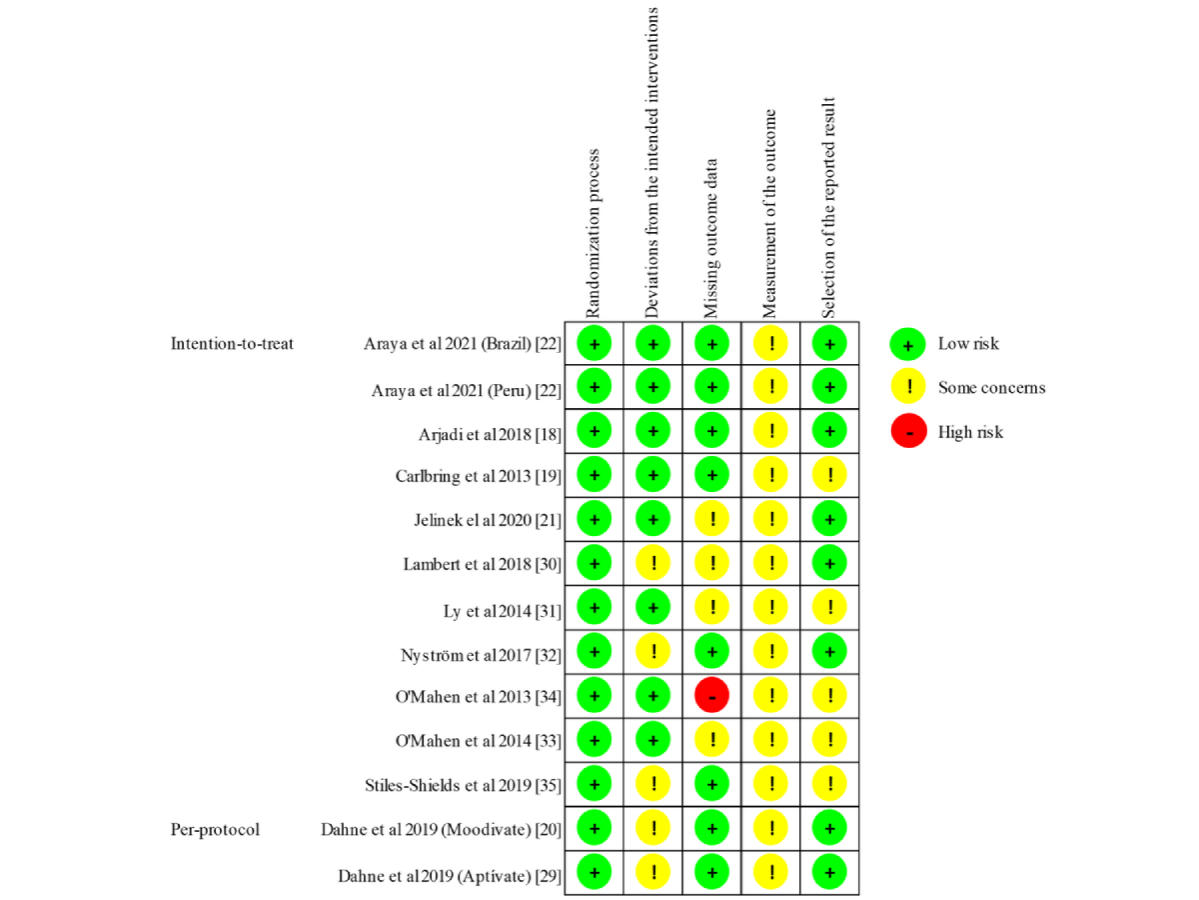
Risk of bias summary.

A visual summary of the risk of bias assessment is displayed in Figure 2. A detailed description of the authors’ judgments can be found in Multimedia Appendix 9. Due to assessment through self-report measures that made blinding impossible, all studies were classified as “with some concerns” (RoB: measurement of the outcome). Some concerns were further raised, if studies could not rule out nonadherence or unequal therapist support (deviations from the intended intervention) [20,29,30,32,35], did not present a preregistration or protocol (selection of the reported result) [19,31,33-35], or could not verify that dropout was not related to participants’ health status (missing outcome data) [21,30,31,33]. A high risk of bias occurred only for 1 study that had a very high dropout rate [34].

### Meta-Analyses of the Main Outcome of Depressive Symptoms

**Figure 3:**
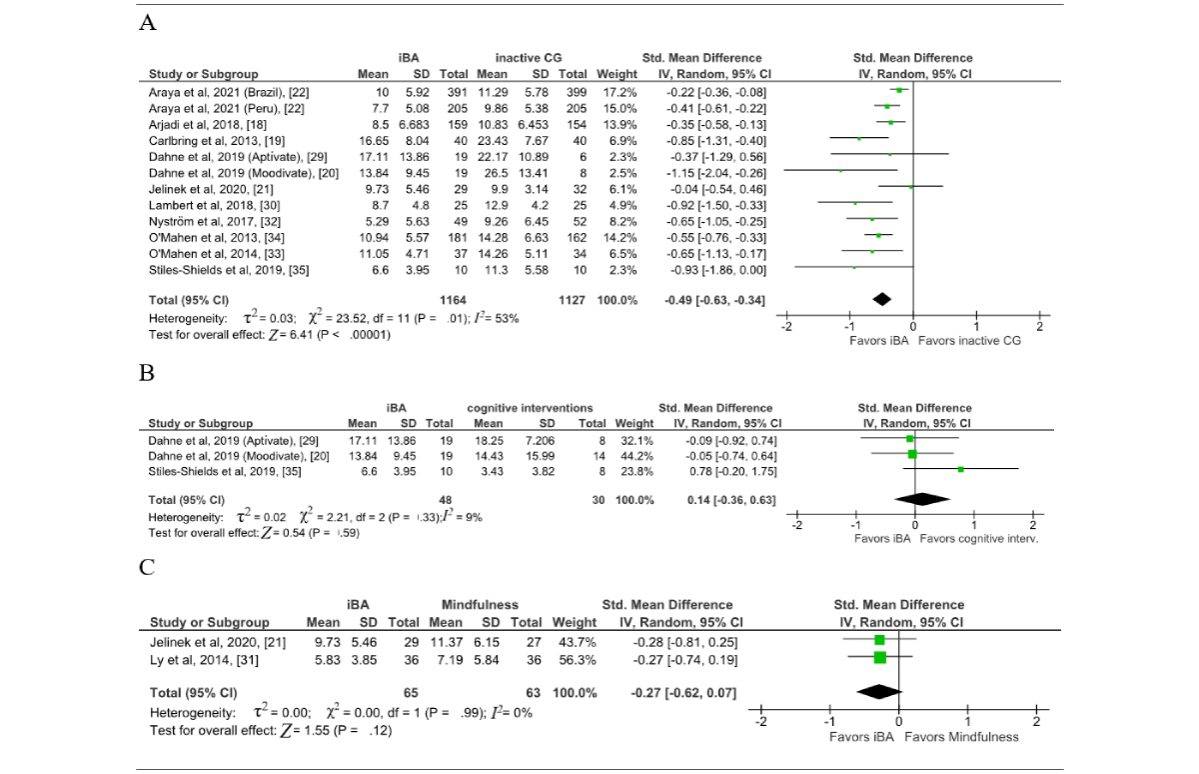
Effects of iBA in comparison to (A) inactive CGs on depression, (B) cognitive intervention on depression, and (C) mindfulness on depression. CG: control group; iBA: internet-based behavioral activation.

Out of the 13 studies included, 12 reported effects of iBA compared to inactive CGs on depressive symptoms and were included in the main analysis [18-22,29,30,32-35]. A statistically significant effect was revealed favoring iBA against inactive CGs for the treatment of depressive symptoms, with an SMD of −0.49 (95% CI −0.63 to −0.34; *P*<.001) (Figure 3). The overall level of heterogeneity was moderate to substantial (*I*^2^=53%). Visual inspection of the funnel plot revealed slight asymmetry at the bottom right, suggesting some publication bias (Multimedia Appendix 10). None of the conducted sensitivity analyses changed the significance of the effect, as the effect size ranged from −0.37 to −0.55 in favor of iBA. Pure iBA interventions showed a slightly smaller effect [18,20-22,29,32,35]. At the 6-month follow-up, there was no significant effect (SMD −0.12; 95% CI −0.27 to 0.04; *P*=.13; Multimedia Appendix 11).

In addition to the main analysis, meta-analyses of iBA compared to cognitive interventions [20,29,35] and iBA compared to mindfulness [21,31] were calculated. There was no significant effect in either of the 2 comparisons (cognitive interventions: SMD 0.14, 95% CI −0.36 to 0.63, *P*=.59; *I*^2^=9%; mindfulness: SMD −0.27, 95% CI −0.62 to 0.07, *P*=.12, *I*^2^=0%; Figure 3), and heterogeneity was low in both cases.

### Subgroup Analyses

**Figure 4:**
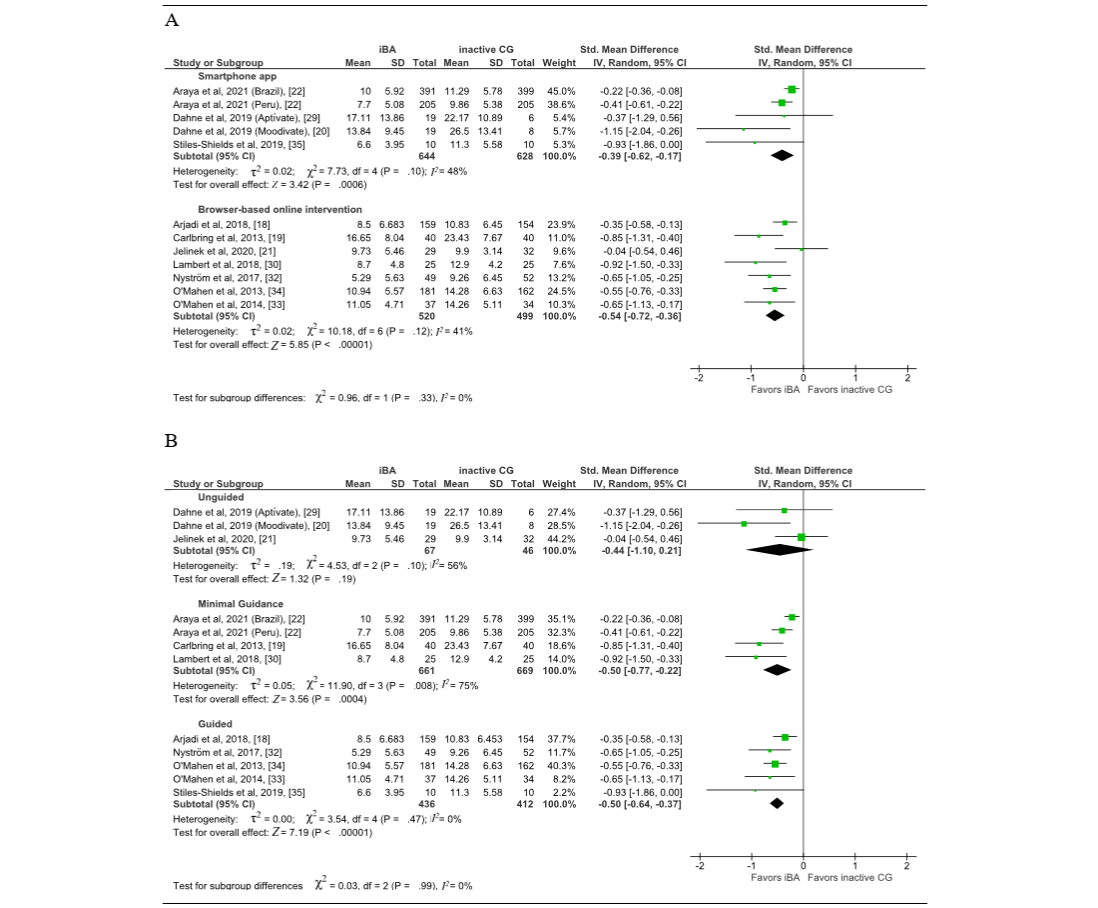
Effects of iBA on depression. Subgroup analyses for (A) mode of delivery and (B) guidance. CG: control group; iBA: internet-based behavioral activation.

Subgroup analyses (Figure 4) and sensitivity analyses (Multimedia Appendix 12) were only performed for the main analysis (depressive symptoms). Studies on browser-based digital interventions [18,19,21,30,32-34] showed slightly higher effects (SMD −0.54; 95% CI −0.72 to −0.36; *P*<.001; *I*^2^=41%) than studies on a smartphone app (SMD −0.39; 95% CI −0.62 to −0.17; *P*<.001; *I*^2^=48%) [20,22,29,35]. The subgroup analysis on the level of guidance revealed significant results only for interventions with minimal guidance (SMD −0.50 95% CI −0.77 to −0.22; *P*<.001) [19,22,30] and guided interventions (SMD −0.50 95% CI −0.64 to −0.37; *P*<.001) [18,32-35], whereas the effect of unguided interventions [20,21,29] did not reach significance. Heterogeneity for interventions with minimal guidance was substantial (*I*^2^=75%) and low (*I*^2^=0%) for guided interventions.

### Meta-Analyses of Secondary Outcomes

Secondary outcome analyses were conducted for anxiety, quality of life, and activation (Multimedia Appendix 13).

In total, 4 studies reported results for the secondary outcome of anxiety symptoms [19,30,32,33]. The meta-analysis on anxiety symptoms showed a statistically significant effect in favor of iBA compared to inactive CGs for the treatment of anxiety (SMD −0.59; 95% CI −0.83 to −0.35; *P*<.001) with low statistical heterogeneity (*I*^2^=0%).

Quality of life was reported by 5 studies [18,19,21,22]. A statistically significant effect was found beneficial to iBA compared to inactive CGs (SMD 0.20; 95% CI 0.10-0.29; *P*<.001), with higher scores indicating higher quality of life. Statistical heterogeneity was low (*I*^2^=0%).

The outcome of activation data from 3 studies could be combined [21,22], which used the Behavioral Activation for Depression Scale [36]. A statistically significant effect favoring iBA compared to inactive CGs was found (SMD 0.25; 95% CI 0.01-0.48; *P*<.05) for activation with higher scores, indicating higher activation. Heterogeneity for this comparison was substantial (*I*^2^=66%).

## Discussion

### Summary of Findings

The present review and meta-analysis summarizes the current state of research in the dynamic field of iBA. Across 12 RCTs, iBA appeared to be more effective than inactive CGs with medium effect sizes. This effect was robust in all conducted sensitivity analyses. Subgroup analyses revealed slightly higher effect sizes for browser-based digital interventions than smartphone apps [37]. Interventions with guidance demonstrated their effectiveness, whereas interventions without guidance did not. Our meta-analysis showed no differences in effectiveness between minimal guidance and more intensive forms of guidance. The investigation of the optimal type and amount of guidance is a pending topic that could make a critical difference concerning the cost-effectiveness and scalability of interventions [38]. The stability of effects could not be found at the 6-month follow-up. Yet, only a very limited number of follow-up data was available. Future research should clarify the long-term effects of iBA.

Regarding our data, the effects of iBA on depressive symptoms seem to be comparable to the effects of internet-based CBT or mindfulness interventions. This finding equals the results of other meta-analyses reporting comparable effect sizes for different treatment approaches [9,10]. BA is associated with several key strengths such as low-cost applicability by nurses and lay counselors [39,40]. It could bridge waiting times and serve as a low-threshold access to psychotherapy.

In our meta-analyses, iBA is also associated with reduced anxiety symptoms and increased quality of life and activation. The effects on anxiety symptoms underline the transdiagnostic potential of BA [41,42]. There is initial evidence that BA is effective in psychotic disorders (reduction of negative symptoms) [43], eating disorders [44], and posttraumatic stress disorders [45].

There was clinical heterogeneity of included interventions regarding settings [29,30], populations [33,34], duration of the interventions [21,33], the number of modules [22,35], and cultural groups [18,22], which suggests a broad field of iBA applications. The mode of intervention delivery of included studies, however, was limited to browser-based interventions and smartphone apps. For this review, we did not identify any iBA interventions involving chatbots, artificial intelligence systems, or just-in-time adaptive interventions. Statistical heterogeneity was moderate to substantial for the main analysis and low to substantial for the secondary outcomes meta-analyses.

### Strengths and Limitations

We have conducted a comprehensive review and meta-analysis in accordance with PRISMA guidelines, including independent ratings, sensitivity analyses, and the assessment of publication bias. An elaborated search term based on former meta-analyses allowed us to conduct a specific search on iBA. The specificity of our search may have led to the nondetection of a few particular studies that did not label their BA interventions as “behavioral activation” or the synonyms we searched for. Yet, the fact that we detected all studies of our validation set supports our approach. We did not search for unpublished research and only included papers in English or German. We were able to sharpen the focus on iBA, excluding interventions that were not predominantly transmitted in a web-based setting (eg, blended care) and that contained other main elements besides BA. The risk of bias rating revealed some concerns for all studies, and there was evidence of possible publication bias. The main concern was missing external assessment of depressive symptomatology, as all included studies in this meta-analysis relied on self-report measures. Interventions varied in their intensity, and many studies lacked a detailed intervention description. For the main analysis, we observed a moderate to substantial statistical heterogeneity. By separating interventions based on their level of guidance, heterogeneity was lower in both subgroups. To reduce heterogeneity in future meta-analyses, it might therefore be useful to consider conducting separate analyses, depending on the level of guidance. The different lengths of interventions also resulted in different postmeasurement time points and may have also contributed to heterogeneity. If in the future more primary studies with different postmeasurement time points are available, these should be grouped to inform about the stability of the effect over time.

### Conclusions

The findings of this study suggest that iBA is effective in reducing depressive symptoms and increasing activation in daily life. It may also reduce anxiety symptoms of individuals with depression and improve their quality of life. There is an enormous potential for iBA interventions in lower-income countries and health care systems with limited therapeutic capacities.

## Appendix

Multimedia Appendix 1 PRISMA checklist.

Multimedia Appendix 2 Amendments and specifications to the PROSPERO registration.

Multimedia Appendix 3 Search strategies.

Multimedia Appendix 4 Validation set.

Multimedia Appendix 5 Inclusion criteria checklist.

Multimedia Appendix 6 List of extracted data.

Multimedia Appendix 7 Levels of guidance.

Multimedia Appendix 8 Detailed intervention description of included studies.

Multimedia Appendix 9 Risk of bias assessment. Authors' judgements.

Multimedia Appendix 10 Funnel plot.

Multimedia Appendix 11 Forest plot of 6-month follow-up.

Multimedia Appendix 12 Forest plot of sensitivity analyses.

Multimedia Appendix 13 Forest plots of secondary outcomes anxiety, quality of life, and activation.

## Abbreviations

BA: behavioral activation
CBT: cognitive behavioral therapy
CG: control group
iBA: internet-based behavioral activation
ITT: intention-to-treat
PRISMA: Preferred Reporting Items for Systematic Reviews and Meta-Analyses
RCT: randomized controlled trial
RoB 2: Cochrane Collaboration’s Risk of Bias tool 2
SMD: standardized mean difference
