# Supplementary material for: Internet-Based Behavioral Activation for Depression: Systematic Review and Meta-Analysis
Source: J Med Internet Res. 2023 May 25;25:e41643. doi: 10.2196/41643 (PMC10251223; doi:10.2196/41643)
Supplement: Multimedia Appendix 2 [file jmir_v25i1e41643_app2.pdf]

Multimedia Appendix 2. Amendments and specifications to the PROSPERO registration

Amendment 1:

In the PROSPERO registration we planned to only include studies with adult samples. In our pre-registration, we did not define if the adult state refers to the inclusion criterion of the original study or its final sample. We had to specify this during the study screening process. Both reviewers independently classified the sample of Arjadi et al. (2018) as adult. There was no other study with mixed or minor samples that was eligible for the meta-analysis.

Reason: The sample represents an adult sample with 97,4% pf the participants aged 18 or older. The mean age of the 313 participants within this study was 24.48 [SD = 5.07]. The sample only included three 16-year-old participants and five 17-year-old participants.

A sensitivity analysis regarding the age of participants was performed.

Amendment 2:

We made a post-hoc decision to calculate a meta-analysis for the follow-up measurement point of 6 months after randomization. We chose the 6-month measurement point to investigate the long-term stability of the effect as data were available for more than two studies. For the follow-up after 1 month, data were only available for two studies (Jelinek 2020, Stiles-Shields 2019). For the 3-month follow-up data were available for one study (Arjadi, 2018), for Carlbring et al. 2013, only single-arm data (IG only) were collected at the 3-month follow-up.

Amendment 3:

Title- and abstract screening was planned to be conducted by two independent reviewers for only 20% of the studies.

Title- and abstract-screening was fully conducted (100%) by two independent reviewers.

Amendment 4:

Two studies, Jelinek et al. 2020 and Ly et al. 2014 did not meet the a priori defined cut-off score for depressive symptoms.

We decided to still include these studies in our analysis and conducted a sensitivity analysis excluding the results of these studies from our meta-analysis to assess the impact of this decision on our result. The effect remained robust.

Amendment 5:

The reported pooled mean age across included studies was conducted without data from O'Mahen et al. 2014, as the needed information was not available in this study.

Amendment 6:

No funding sources were pre-registered. We received funding for the article processing by the Open Access Publication Fund of the University of Freiburg. Further, this review and meta-analysis was made possible by funding from the German Research Foundation (Deutsche Forschungsgemeinschaft, grant number: 425987318) and by institutional budget of the University of Freiburg, Institute of Psychology.
