# Supplementary material for: Internet-Based Behavioral Activation for Depression: Systematic Review and Meta-Analysis
Source: J Med Internet Res. 2023 May 25;25:e41643. doi: 10.2196/41643 (PMC10251223; doi:10.2196/41643)
Supplement: Multimedia Appendix 3 [file jmir_v25i1e41643_app3.pdf]

Multimedia Appendix 3. Search strategies

Search Strategy for EBSCO (Medline, PsychINFO, PSYNDEX)

S1	TI ehealth* OR AB ehealth* OR KW ehealth* OR SU ehealth*
S2	TI e-health* OR AB e-health* OR KW e-health* OR SU e-health*
S3	TI telehealth* OR AB telehealth* OR KW telehealth* OR SU telehealth*
S4	TI telemedicine* OR AB telemedicine* OR KW telemedicine* OR SU telemedicine*
S5	TI tele-based* OR AB tele-based* OR KW tele-based* OR SU tele-based*
S6	TI digital* OR AB digital* OR KW digital* OR SU digital*
S7	TI internet* OR AB internet* OR KW internet* OR SU internet*
S8	TI online* OR AB online* OR KW online* OR SU online*
S9	TI web* OR AB web* OR KW web* OR SU web*
S10	TI computer* OR AB computer* OR KW computer* OR SU computer*
S11	TI mobile* OR AB mobile* OR KW mobile* OR SU mobile*
S12	TI smartphone* OR AB smartphone* OR KW smartphone* OR SU smartphone*
S13	TI smart phone* OR AB smart phone* OR KW smart phone* OR SU smart phone*
S14	TI cell* phone* OR AB cell* phone* OR KW cell* phone* OR SU cell* phone*
S15	TI cellphone* OR AB cellphone* OR KW cellphone* OR SU cellphone*
S16	TI instant messag* OR AB instant messag* OR KW instant messag* OR SU instant messag*
S17	TI text messag* OR AB text messag* OR KW text messag* OR SU text messag*
S18	TI telerehabilitation* OR AB telerehabilitation* OR KW telerehabilitation* OR SU telerehabilitation*
S19	TI telecommunication* OR AB telecommunication* OR KW telecommunication* OR SU telecommunication*
S20	TI remote* consultation* OR AB remote* consultation* OR KW remote* consultation* OR SU remote* consultation*
S21	TI electronic* health* services* OR AB electronic* health* services* OR KW electronic* health* services* OR SU electronic* health* services*
S22	TI telepsychology* OR AB telepsychology* OR KW telepsychology* OR SU telepsychology*
S23	S1 OR S2 OR S3 OR S4 OR S5 OR S6 OR S7 OR S8 OR S9 OR S10 OR S11 OR S12 OR S13 OR S14 OR S15 OR S16 OR S17 OR S18 OR S19 OR S20 OR S21 OR S22
S24	TI behavio* activat* OR AB behavio* activat* OR KW behavio* activat* OR SU behavio* activat*
S25	TI self monitor* OR AB self monitor* OR KW self monitor* OR SU self monitor*
S26	TI activity schedul* OR AB activity schedul* OR KW activity schedul* OR SU activity schedul*
S27	TI activity monitor* OR AB activity monitor* OR KW activity monitor* OR SU activity monitor*
S28	TI ((pleas* OR enjoyable OR rewarding) (activit* OR event*)) OR AB ((pleas* OR enjoyable OR rewarding) (activit* OR event*)) OR KW ((pleas* OR enjoyable OR rewarding) (activit* OR event*)) OR SU ((pleas* OR enjoyable OR rewarding) (activit* OR event*))
S29	S24 OR S25 OR S26 OR S27 OR S28
S30	TI depress* OR AB depress* OR KW depress* OR SU depress*
S31	TI mood disorder* OR AB mood disorder* OR KW mood disorder* OR SU mood disorder*
S32	TI affective disorder* OR AB affective disorder* OR KW affective disorder* OR SU affective disorder*
S33	TI dysthym* OR AB dysthym* OR KW dysthym* OR SU dysthym*
S34	S30 OR S31 OR S32 OR S33

S35	TI RCT* OR AB RCT* OR KW RCT* OR SU RCT*
S36	TI random* OR AB random* OR KW random* OR SU random*
S37	TI clinical* trial* OR AB clinical* trial* OR KW clinical* trial* OR SU clinical* trial*
S38	S35 OR S36 OR S37
S39	S23 AND S29 AND S34 AND S38

Search Strategy for CENTRAL

#1	(ehealth*):ti,ab,kw
#2	(e-health*):ti,ab,kw
#3	(telehealth*):ti,ab,kw
#4	(telemedicine*):ti,ab,kw
#5	(telebased*):ti,ab,kw
#6	(tele-based*):ti,ab,kw
#7	(digital*):ti,ab,kw
#8	(internet*):ti,ab,kw
#9	(online*):ti,ab,kw
#10	(web*):ti,ab,kw
#11	(computer*):ti,ab,kw
#12	(mobile*):ti,ab,kw
#13	(smartphone*):ti,ab,kw
#14	(smart phone*):ti,ab,kw
#15	(cell* phone*):ti,ab,kw
#16	(cellphone*):ti,ab,kw
#17	(instant messag*):ti,ab,kw
#18	(text messag*):ti,ab,kw
#19	(telerehabilitation*):ti,ab,kw
#20	(telecommunication*):ti,ab,kw
#21	(remote* consultation*):ti,ab,kw
#22	(electronic* health* services*):ti,ab,kw
#23	(telepsychology*):ti,ab,kw
#24	MeSH descriptor: [Telemedicine] this term only
#25	MeSH descriptor: [Digital Technology] this term only
#26	MeSH descriptor: [Internet] this term only
#27	MeSH descriptor: [Computers] this term only
#28	MeSH descriptor: [Smartphone] this term only
#29	MeSH descriptor: [Cell Phone] this term only
#30	MeSH descriptor: [Telecommunications] this term only
#31	MeSH descriptor: [Communications Media] this term only

#32	MeSH descriptor: [Text Messaging] this term only
#33	MeSH descriptor: [Telerehabilitation] this term only
#34	MeSH descriptor: [Remote Consultation] this term only
#35	#1 OR #2 OR #3 OR #4 OR #5 OR #6 OR #7 OR #8 OR #9 #10 OR #11 OR #12 OR #13 OR #14 OR #15 OR #16 OR #17 OR #18 OR #19 OR #20 OR #21 OR #22 OR #23 OR #24 OR #25 OR #26 OR #27 OR #28 OR #29 OR #30 OR #31 OR #32 OR #33 OR #34
#36	(behavio* activat*):ti,ab,kw
#37	(self monitor*):ti,ab,kw
#38	(activity schedul*):ti,ab,kw
#39	((pleas* OR enjoyable OR rewarding) (activit* OR event*)):ti,ab,kw
#40	#36 OR #37 OR #38 OR #39
#41	(depress*):ti,ab,kw
#42	(mood disorder*):ti,ab,kw
#43	(affective disorder*):ti,ab,kw
#44	(dysthym*):ti,ab,kw
#45	MeSH descriptor: [Depression] this term only
#46	MeSH descriptor: [Mood Disorders] this term only
#47	MeSH descriptor: [Affective Symptoms] this term only
#48	MeSH descriptor: [Dysthymic Disorder] this term only
#49	MeSH descriptor: [Depressive Disorder] this term only
#50	#41 OR #42 OR #43 OR #44 OR #45 OR #46 OR #47 OR #48 OR #49
#51	(RCT*):ti,ab,kw
#52	(random*):ti,ab,kw
#53	(clinical* trial*):ti,ab,kw
#54	MeSH descriptor: [Random Allocation] this term only
#55	MeSH descriptor: [Randomized Controlled Trial] this term only
#56	MeSH descriptor: [Clinical Trial] this term only
#57	#51 OR #52 OR #53 OR #54 OR #55 OR #56
#58	#35 AND #40 AND #50 AND #57
