# Supplementary material for: Internet-Based Behavioral Activation for Depression: Systematic Review and Meta-Analysis
Source: J Med Internet Res. 2023 May 25;25:e41643. doi: 10.2196/41643 (PMC10251223; doi:10.2196/41643)
Supplement: Multimedia Appendix 4 [file jmir_v25i1e41643_app4.pdf]

Multimedia Appendix 4. Validation Set

Sources: Previous meta-analyses on behavioral activation (Uphoff et al. 2020) and internet-based behavioral activation (Huguet et al. 2018).

- Huguet A, Miller A, Kisely S, Rao S, Saadat N, McGrath PJ. A systematic review and meta-analysis on the efficacy of Internet-delivered behavioral activation. *J Affect Disord*. 2018;235:27-38. Doi:10.1016/j.jad.2018.02.073
- Uphoff E, Ekers D, Robertson L, et al. Behavioural activation therapy for depression in adults. *Cochrane Database Syst Rev*. 2020;7:CD013305. Doi:10.1002/14651858.CD013305.pub2

No. [Reference No.]	Study	Source
1 [19]	Carlbring P, Hägglund M, Luthström A, et al. Internet-based behavioral activation and acceptance-based treatment for depression: a randomized controlled trial. *J Affect Disord*. 2013;148(2-3):331-337. doi:10.1016/j.jad.2012.12.020	Huguet et al. 2018
2 [31]	Ly KH, Trüschel A, Jarl L, et al. Behavioural activation versus mindfulness-based guided self-help treatment administered through a smartphone application: a randomised controlled trial. *BMJ Open*. 2014;4(1):e003440. doi:10.1136/bmjopen-2013-003440	Huguet et al. 2018
3 [32]	Nyström MBT, Stenling A, Sjöström E, et al. Behavioral activation versus physical activity via the internet: A randomized controlled trial. *J Affect Disord*. 2017;215:85-93. doi:10.1016/j.jad.2017.03.018	Huguet et al. 2018
4 [34]	O'Mahen HA, Woodford J, McGinley J, et al. Internet-based behavioral activation--treatment for postnatal depression (Netmums): a randomized controlled trial. *J Affect Disord*. 2013;150(3):814-822. doi:10.1016/j.jad.2013.03.005	Huguet et al. 2018
5 [33]	O'Mahen HA, Richards DA, Woodford J, et al. Netmums: a phase II randomized controlled trial of a guided Internet behavioural activation treatment for postpartum depression. *Psychol Med*. 2014;44(8):1675-1689. doi:10.1017/S0033291713002092	Huguet et al. 2018
6 [18]	Arjadi R, Nauta MH, Scholte WF, et al. Internet-based behavioural activation with lay counsellor support versus online minimal psychoeducation without support for treatment of depression: a randomised controlled trial in Indonesia. *The Lancet Psychiatry*. 2018;5(9):707-716. doi:10.1016/S2215-0366(18)30223-2	Uphoff et al. 2020
7 [35]	Stiles-Shields C, Montague E, Kwasny MJ, Mohr DC. Behavioral and cognitive intervention strategies delivered via coached apps for depression: Pilot trial. *Psychol Serv*. 2019;16(2):233-238. doi:10.1037/ser0000261	Uphoff et al. 2020
