# Supplementary material for: Internet-Based Behavioral Activation for Depression: Systematic Review and Meta-Analysis
Source: J Med Internet Res. 2023 May 25;25:e41643. doi: 10.2196/41643 (PMC10251223; doi:10.2196/41643)
Supplement: Multimedia Appendix 5 [file jmir_v25i1e41643_app5.pdf]

Multimedia Appendix 5. Inclusion criteria checklist

**General Characteristics**	yes	no
1. Is the record an original research article (not only a trial registration)?	☐	☐
2. Is the record written in English or German?	☐	☐
3. Is the record not a study protocol?	☐	☐
4. Is the record a randomized controlled trial (RCT)?	☐	☐
	☐	☐
**Population**		
5. Does the record assess a population which suffers from clinically relevant depression?	☐	☐
Depressive symptoms above cut-off before study inclusion (major depression, chronic major depression, dysthymia, double depression, recurrent depression without complete remission between episodes, depressive symptoms)		
Recorded by self-rating scales or clinical ratings	☐	☐
	☐	☐
6. Is the population an adult sample (< 18 years)?		
	☐	☐
**Intervention**		
7. Is the conducted intervention Behavioral Activation? (= Intervention that focusses on increasing patients' daily activities and access to reinforcement)	☐	☐
• Stand-alone intervention or no other main component (cognitive treatment, combinations with other treatments (PST, ACT)	☐	☐
8. Is the intervention delivered via internet? (Computer-based, smartphone intervention, mobile app, online tool, …)	☐	☐
• No videoconference, face-to-face therapy, or telephone-based therapy as main element	☐	☐
**Comparator**		
9. No restrictions		
**Outcome**		
10. Does the record contain clinical outcome data for depressive symptomatology (via BDI, PHQ-9, HDRS, …)	☐	☐
**Further criteria**		
11. Is the record an original research article?	☐	☐
12. Is the record not only a secondary analysis of existing data from previous studies?	☐	☐
