# Supplementary material for: Internet-Based Behavioral Activation for Depression: Systematic Review and Meta-Analysis
Source: J Med Internet Res. 2023 May 25;25:e41643. doi: 10.2196/41643 (PMC10251223; doi:10.2196/41643)
Supplement: Multimedia Appendix 6 [file jmir_v25i1e41643_app6.pdf]

Multimedia Appendix 6. List of extracted data

- publication of the study: title, authors, year of publication, country of trial implementation
- population: age, sex, and diagnosis or treated condition of participants, comorbid condition(s)
- intervention(s): delivery method, intervention content, duration, BA-model used, amount of guidance
- comparator(s): number of arms, type of comparison group(s)
- study design: study setting, post- and follow-up measures, length of follow-up(s)
- outcome(s): outcome measures, scales, number of cases, mean and standard deviation for each continuous outcome and assessment time point
- study characteristics: number of participants separately per group and measure time point, total dropout
