# Supplementary material for: Internet-Based Behavioral Activation for Depression: Systematic Review and Meta-Analysis
Source: J Med Internet Res. 2023 May 25;25:e41643. doi: 10.2196/41643 (PMC10251223; doi:10.2196/41643)
Supplement: Multimedia Appendix 7 [file jmir_v25i1e41643_app7.pdf]

Multimedia Appendix 7. Levels of guidance

Unguided interventions:

Interventions are labeled as unguided interventions, if study participants did not receive technical, therapeutic or any other support throughout the intervention besides a quick explanation of how to use the intervention app/computer program at the beginning of the intervention before usage. There was no help regarding clinical issues and no therapeutic contact.

Interventions with minimal guidance:

Interventions are labeled as having minimal guidance, if support throughout the intervention was of technical nature or was implemented only if non-adherence occurred to enhance motivation to use the intervention. Help regarding clinical issues or therapeutic contact was not present or not obligatory and of minimal dimension only, meaning indirect contact (via messaging service or internet) not exceeding 15 min per week.

Guided interventions:

Interventions were considered as guided interventions, if study participants received help with clinical issues throughout the interventions and had direct therapeutic support via phonecalls, chat rooms, or other direct contact possibilities or indirect help with clinical issues or therapeutic contact that was given on regular basis.
