# Supplementary material for: Internet-Based Behavioral Activation for Depression: Systematic Review and Meta-Analysis
Source: J Med Internet Res. 2023 May 25;25:e41643. doi: 10.2196/41643 (PMC10251223; doi:10.2196/41643)
Supplement: Multimedia Appendix 8 [file jmir_v25i1e41643_app8.pdf]

Multimedia Appendix 8: Detailed intervention description of included studies

First author, year	Delivery mode	Name of intervention	Country	Duration of treatment, weeks	Number of modules/sessions (time effort per module/session)	Compliance	Approximate time per session, minutes	Intervention components (underlying BA manual)	Guidance
Araya et al, 2021 [22] (Brazil)	Smartphone app	CONEMO	Brazil	6	18 (10 min)	18% did not complete any active intervention sessions; 65% completed 9 or more sessions: 45% completed all 18 sessions	10	BA (NA)	Minimal guidance
Araya et al, 2021[22] (Peru)	Smartphone app	CONEMO	Peru	6	18 (10 min)	2% did not complete any active intervention sessions; 92% completed 9 or more sessions, 78% completed all 18 sessions	10	BA (NA)	Minimal guidance
Arjadi et al, 2018 [18]	Online program	Guided Act and Feel Indonesia	Indonesia	8	8 (30-45 min)	Mean log in to program: 13.93 times [SD= 10.92] Mean module completion: 5	30-45	BA (Lewinsohn)	Guided
Carlbring et al, 2013 [19]	Online program	Depressionshjälpen	Sweden	8	7 (NA)	Mean module completion: 5.1 [SD= 1.7] Time spent in total: 270 min	NA	BA[a] (Kanter), ACT[b]	Minimal guidance
Dahne et al, 2019 [20] (Moodivate)	Smartphone app	Moodivate	United States	8	NA	Time spent in total within the app: 120.76 min [101.02]	NA	BA (Lejuez)	Unguided
Dahne et al, 2019 [29] (Aptívate)	Smartphone app	Aptivate	United States	8	NA	Time spent in total within the app: 65.77 min [SD=82.76]	NA	BA (Lejuez)	Unguided
Jelinek et al, 2020 [21]	Web-based intervention	NA	Germany	2	Daily advised usage (60 min)	Lack of use of the intervention at t1: iBA: 6 participants (27, 22%) iMBI: 3 participants (25, 12%) Lack of information on	60	BA (Martell)	Unguided

						usage due to technical difficulties:<br>iBA: 2 participants<br>iMBI:2 participants			
Lambert et al, 2018 [30]	Web-based intervention	eMotion	United Kingdom	8	13 (NA)	Modules accessed: 3 [IQR 2-5]<br>Total time spent: 41.3 min [IQR 18.9-90.4]	NA	BA[a] (NA), PST[b]	Minimal guidance
Ly et al, 2014 [31]	Smartphone app	NA	Sweden	8	NA	IBA: 63% adherence rate<br>Mindfulness: 78 % adherence rate	NA	BA (Martell and Lejuez)	Guided
Nyström et al, 2017 [32]	Web-based intervention	NA	Sweden	12	8 (NA)	NA	NA	BA (Martell and Lejuez)	Guided
O'Mahen et al, 2013 [34]	Online program	Postnatal iBA	United Kingdom	15	11 (40 min)	NA	40	BA[a] (Martell), Rumination[b]	Guided
O'Mahen et al, 2014 [33]	Online program	NetmumsHWD	United Kingdom	17	12 (29 min)	Mean module completion: 5.36 [SD= 4.62]<br>Total time spent: 253 min	30	BA[a] (Martell), Rumination[b]	Guided
Stiles-Shields et al, 2019 [35]	Smartphone app	Boost Me	United States	6	6 (NA)	App launches: average of 100/6 weeks	NA	BA (Martell)	Guided

Abbreviations: ACT, acceptance commitment therapy; BA, behavioral activation; CONEMO, emotional control; IQR, interquartile range; NA, not available; PST, problem solving therapy; SD, standard deviation.

[a] Main component
[b] Additional minimal component
