# Supplementary material for: Internet-Based Behavioral Activation for Depression: Systematic Review and Meta-Analysis
Source: J Med Internet Res. 2023 May 25;25:e41643. doi: 10.2196/41643 (PMC10251223; doi:10.2196/41643)
Supplement: Multimedia Appendix 9 [file jmir_v25i1e41643_app9.pdf]

Multimedia Appendix 9. Risk of bias assessment: Authors' judgments

Study: Araya et al, 2021 [22] (Brazil)

Bias	Authors' judgment	Support for judgment
Randomization process	Low risk of bias	Quote: "In São Paulo, the sample was stratified according to teaching status, with a single block in each stratum. A statistician, who was not involved in recruitment, undertook the cluster, stratified randomization of clinics blind to their identities, with 10 clinics randomized to each group, 5 from each stratum."
Deviations from intended interventions	Low risk of bias	Quote: 'Research assistants collecting baseline and outcome data from participants were blind to treatment allocation. Concealing allocation from clinical staff delivering the digital intervention or managing the safety net was not feasible.'<br><br>Judgment comment: No specific information was found concerning deviations from the intended intervention, but precautions were taken to prevent the bias.
Missing outcome data	Low risk of bias	Judgment comment: Dropout of 15 percent (N=375/440) in the intervention group and (N=379/440) in the control group was reported. However, the authors provided evidence that the result was not biased by missing outcome data.
Measurement of the outcome	Some concerns	Judgment comment: The PHQ-9 as an appropriate measurement was used to assess the outcome at comparable time points for both groups. Outcome assessment was by self-report. Study participants were not blinded to treatment allocation. Knowledge of the assigned intervention could have influenced participant-reported outcomes.
Selection of the reported result	Low risk of bias	Judgment comment: The trial protocol with a detailed statistical analysis plan is available (https://clinicaltrials.gov/ct2/show/study/ NCT02846662?term=02846662&draw=2&rank=1). The authors provided specific changes and reasons for changes.
Overall	Some concerns	

Study: Araya et al, 2021 [22] (Peru)

Bias	Authors' judgment	Support for judgment
Randomization process	Low risk of bias	Quote: 'In Lima, individual randomization was undertaken using a 1:1 allocation ratio, with balance attained with respect for any of the countries health centre and baseline severity of depressive symptoms (PHQ-9 score <15 or ≥15) through stochastic minimization with a 30% chance of simple random allocation, using an online randomization system.'
Deviations from intended interventions	Low risk of bias	Quote: 'Research assistants collecting baseline and outcome data from participants were blind to treatment allocation. Concealing allocation from clinical staff delivering the digital

		intervention or managing the safety net was not feasible.'<br><br>Judgment comment: Due to cluster randomization, participants probably weren't aware of their assigned intervention. No specific information was found concerning deviations from the intended intervention, but precautions were taken to prevent the bias.
Missing outcome data	Low risk of bias	Judgment comment: Nearly all participants' outcome data were available.
Measurement of the outcome	Some concerns	Judgment comment: The method of measuring the outcome was appropriate and identical for both groups. The outcome assessor was the study participant, who was aware of his allocation. Knowledge of the assigned intervention could have influenced participant-reported outcomes.
Selection of the reported result	Low risk of bias	Judgment comment: Trial protocol with a detailed statistical analysis plan is available (https://clinicaltrials.gov/ct2/show/NCT03026426?term =03026426&draw=2&rank=1). The authors provided specific changes and reasons for changes.
Overall	Some concerns	

Study: Arjadi et al, 2018 [18]

Bias	Authors' judgment	Support for judgment
Randomization process	Low risk of bias	Quote: 'Participants were randomly allocated (1:1) by a research assistant via a web-based randomisation program built by an independent developer for this trial.'
Deviations from intended interventions	Low risk of bias	Quote: 'The research assistants who did the clinical interviews after randomisation were not involved in the intervention process and were masked to participants' treatment condition (participants were also asked not to reveal their treatment condition during the interviews).'<br><br>Judgment comment: Participants were not blind to their treatment allocation. However, there were no deviations from the intended intervention and an ITT analysis was used to estimate the effect of assignment to intervention.
Missing outcome data	Low risk of bias	Quote: 'We analysed longitudinal within-participant data with missing values by linear mixed modelling because this method incorporates all available data to prevent complete-case bias.'<br><br>Judgment comment: 39 (25%) participants dropped out of the GAF-ID group and nine (6%) from the control group.
Measurement of the outcome	Some concerns	Judgment comment: An appropriate method was used to assess the outcome at comparable time points for the intervention and the control group. For participant-reported outcomes, the assessment of outcome is potentially influenced by knowledge of intervention received, leading to a judgement of at least 'Some concerns'.
Selection of the reported result	Low risk of bias	Judgment comment: The data were analysed in accordance with the study protocol. The outcome measurements were

		pre-defined questionnaires and it is unlikely that the results have been selected based on the results from multiple eligible analyses of the data.
Overall	Some concerns	

Study: Carlbring et al, 2013 [19]

**Bias**	**Authors' judgment**	**Support for judgment**
Randomization process	Low risk of bias	Quote: 'The participants were divided into two groups–treatment or control–by an online true random-number service independent of the investigators and therapists.'
Deviations from intended interventions	Low risk of bias	Judgment comment: Participants and therapists were not blind to participants' allocation. The authors reported deviations from intended interventions, but exclusion of the affected participants did not result in statistically significant change. Intention-to-treat analysis was used to estimate the effect of assignment to intervention.
Missing outcome data	Low risk of bias	Judgment comment: Only 2,5% of dropouts were reported post-treatment.
Measurement of the outcome	Some concerns	Quote: 'All questionnaires have demonstrated good psychometric properties, even when administered online (Carlbring et al., 2007; Holländare et al.,2010).'<br><br>Judgment comment: Outcome data were assessed at the same time points for both conditions. The outcome assessor was the study participant who was aware of his allocation. Knowledge of the assigned intervention could have influenced participant-reported outcomes.
Selection of the reported result	Some concerns	Judgment comment: The study protocol was mentioned but could not be found. It is unlikely that the numerical result has been selected based on the results from multiple eligible outcome measurements. As to available data, there were no multiple analyses of the data.
Overall	Some concerns	

Study: Dahne et al, 2019 [29] Aptivate

**Bias**	**Authors' judgment**	**Support for judgment**
Randomization process	Low risk of bias	Quote: 'All study procedures were approved by the Medical University of South Carolina Institutional Review Board. Upon consent, participants completed self-report assessments and were subsequently randomized 2:1:1 to receive either the Spanish language Behavioral Activation mobile app (¡Aptívate!), an active control Spanish language Cognitive Behavioral Therapy (CBT) mobile app (iCouch CBT), or Treatment as Usual (i.e., no app).'
Deviations from intended interventions	Some concerns	Judgment comment: Participants were not blind to their assigned intervention. Some participants did not adhere as planned to the intervention. Self-reported app utilization lowered over the weeks (figure 2).

Missing outcome data	Low risk of bias	Judgment comment: Only two participants could not be included in the analysis.
Measurement of the outcome	Some concerns	Judgment comment: The method of measuring the outcome was appropriate. However, the outcome assessor was the study participant, who was not blind to his allocation. Knowledge of the assigned intervention could have influenced participant-reported outcomes.
Selection of the reported result	Low risk of bias	Judgment comment: The study protocol and app development were described previously. The BDI-II (Spanish version) was the only measure for depressive symptoms throughout the study.
Overall	Some concerns	

Study: Dahne et al, 2019 [20] Moodivate

**Bias**	**Authors' judgment**	**Support for judgment**
Randomization process	Low risk of bias	Quote: 'All study procedures were approved by the MUSC IRB. Upon consent, participants completed self-report and interviewer-administered assessments and were subsequently randomized 2:2:1 to receive either the Behavioral Activation mobile app (Moodivate), an active control Cognitive Behavioral Therapy (CBT) mobile app (MoodKit), or Treatment as Usual (i.e., no app).'
Deviations from intended interventions	Some concerns	Quote: 'All participants used the app at least once during the trial, 71.4% of participants used the app at least 28 times, and 42.9% of participants used the app more than 56 times (i.e., at least once per day on average).'<br><br>Judgment comment: Participants were aware of their treatment allocation. Some participants did not adhere as planned to the intervention which could have affected participants´ outcomes.
Missing outcome data	Low risk of bias	Judgment comment: 22 out of 24 participants from the intervention group, all 18 participants from the active control group and all 9 participants from the inactive control group could be included in the analysis.
Measurement of the outcome	Some concerns	Judgment comment: The measurement of the outcome was appropriate and was used for each group at comparable time points. However, the use of self-reported measurements could have influenced the reported outcomes.
Selection of the reported result	Low risk of bias	Judgment comment: The study protocol, app development and testing are available. The BDI was the only measurement for depressive symptoms, that was used continuously throughout the study.
Overall	Some concerns	

Study: Jelinek et al, 2020 [21]

**Bias**	**Authors' judgment**	**Support for judgment**
Randomization	Low risk of bias	Quote: 'Group allocation was performed by a person who did

process		not possess any other information on the respective participant. Participants were randomized at t0 to one of the 3 groups, with an allocation ratio of 1:1:1 based on a fixed randomization plan conducted by a statistician.'
Deviations from intended interventions	Low risk of bias	Judgment comment: Participants received information after the randomization process and were therefore aware of their allocations. The authors reported no deviations from the intended intervention. An appropriate ITT analysis was used to estimate the effect of assignment to intervention.
Missing outcome data	Some concerns	Quote: 'In the ITT group (N=104), 88 participants (85%) completed the t_1 assessment, and 79 (76%) completed the t_2 assessment.'<br><br>Judgment comment: The loss of follow up data could be related to participants' health status.
Measurement of the outcome	Some concerns	Judgment comment: The method of measuring the outcome was appropriate and similar for each group. It cannot be ruled out that treatment allocation influenced outcome responses, especially as there were two active conditions and one waitlist control group and outcome measures were by self-report.
Selection of the reported result	Low risk of bias	Quote: 'Finally, due to recruitment difficulties with the initial study protocol involving the use of the program in an outpatient clinic to bridge waiting times, changes in the trial protocol with regard to recruitment sources were unavoidable, and this also needs to be considered.'<br><br>Judgment comment: The study protocol is available. The numerical result is based on the usage of pre-defined standardized questionnaires.
Overall	Some concerns	

Study: Lambert et al, 2018 [30]

**Bias**	**Authors' judgment**	**Support for judgment**
Randomization process	Low risk of bias	Quote: 'Once participants completed the baseline assessment, they were randomly allocated to either the intervention or control group using simple randomization at the individual level in a 1:1 ratio and a Web-based randomization service (Sealed Envelope Ltd. 2016).'
Deviations from intended interventions	Some concerns	Quote: 'Due to limited resources for the study, the lead author was not blinded to which condition each participant was allocated following randomization. Due to the nature of the intervention, it was also impossible to blind participants to group allocation.'<br><br>Judgment comment: There was non-adherence to the assigned intervention regimen that could have affected participants' outcomes.
Missing outcome data	Some concerns	Judgment comment: 15,6 percent of outcome data were missing from the waitlist control group and 16,6 percent from the intervention group. Reasons for missing data could not

		be found.
Measurement of the outcome	Some concerns	Judgment comment: The PHQ-8 was used to measure the outcome at the same time points in both groups. However, self-reported measurements were used.
Selection of the reported result	Low risk of bias	Judgment comment: The study protocol is available and was followed. The PHQ-8 was the only measure used to assess depressive symptoms. Furthermore, there is evidence that reported results for the outcome measurement correspond to the intended analyses.
Overall	Some concerns	

Study: Ly et al, 2014 [31]

**Bias**	**Authors' judgment**	**Support for judgment**
Randomization process	Low risk of bias	Quote: 'After the recruitment, the participants were allocated using an online randomisation tool (http:// www.random.org), handled by an independent person who was separate from the staff conducting the study.'
Deviations from intended interventions	Low risk of bias	Judgment comment: Participants and therapists were not blind to participants' allocation. No deviations from the intended intervention were reported. The authors used an appropriate analysis to estimate the effect of assignment to intervention.
Missing outcome data	Some concerns	Quote: 'Of the 84 participants randomised, 3 decided not to participate in the study. Nine of these 81 participants (11.1%) did not provide post-treatment data with a distribution of four participants from the BA group and five participants from the mindfulness group.' 'In the 6-month follow-up, 69 participants from the two treatment groups (totalling 85.2%) provided data on the self-report measures, with a distribution of 35 participants from the BA group and 34 participants from the mindfulness group.'<br><br>Judgment comment: The loss of participants' outcome data may be related to participants' health status.
Measurement of the outcome	Some concerns	Judgment comment: The use of self-reported measurements could have influenced the participant-reported outcomes. Given that both groups received an active intervention, it might be less likely that the intervention group specifically influenced the outcome. If one group did receive a treatment while the other group didn't, this would be much more likely.
Selection of the reported result	Some concerns	Judgment comment: There is no information of a study protocol that was specified beforehand. However, pre-defined standardized questionnaires were used.
Overall	Some concerns	

Study: Nyström et al, 2017 [32]

**Bias**	**Authors' judgment**	**Support for judgment**
Randomization	Low risk of bias	Quote: 'Of the 1179 who responded, 312 were considered

process		suitable for participation and were then block randomized independently of the research team (by the project administrator), using a specially designed computer program, into one of the five groups (four treatment groups and one control group).'
Deviations from intended interventions	Some concerns	Judgment comment: Due to the nature of this study, blinding was not possible. There were deviations from the intended intervention that could have affected the outcome. Conductance according to a pre-defined protocol.
Missing outcome data	Low risk of bias	Quote: '312 Participants were randomized. Of these, 286 completed at least one weekly measure or posttreatment and hence could be included in the analyses.<br>The intention-to-treat principle was employed (Streiner, 2002; Streiner and Geddes, 2001), that is, all participants who provided data for at least one weekly measure or the post treatment evaluation were included in the analyses and the full information maximum likelihood (FIML) estimation was used to handle missing data based on the assumption that the data was missing at random (MAR; Enders, 2010).'
Measurement of the outcome	Some concerns	Judgment comment: The method of measuring the outcome was appropriate. At least for the waitlist control group, it is highly probable, that the outcome measure was influenced by being part of this group compared to being part of one of the active groups.
Selection of the reported result	Low risk of bias	Judgment comment: The study protocol is available, and the study was conducted according to plan. Pre-defined and standardized questionnaires were used.
Overall	Some concerns	

Study: O´Mahen et al, 2013 [34]

**Bias**	**Authors' judgment**	**Support for judgment**
Randomization process	Low risk of bias	Quote: 'We randomized consenting participants to the intervention or a treatment as usual (TAU) waiting list control condition remotely using a computer generated code to ensure allocation concealment.'
Deviations from intended interventions	Low risk of bias	Judgment comment: Due to the nature of this study, blinding was not possible. No deviations from the intended intervention took place. An appropriate analysis was used to estimate the effect of assignment to intervention.
Missing outcome data	High risk of bias	Quote: 'In this context, with very high levels of attrition, the MAR assumption cannot be assumed to hold, as propensity for missingness of a patient's EPDS score at week 15 may be related to her (unknown) EPDS score at week 15.'<br><br>Judgment comment: It is difficult to understand which people were assessed for what. Randomization took place before baseline assessment and only about 30% of people randomized completed baseline assessment. More people were included in the analysis that completed baseline assessment. This could be because there was a short and a long version of the baseline assessment and people were

		only counted as 'completed baseline assessment' if they completed the long version. It seems like different analyses were conducted in different subgroups.
Measurement of the outcome	Some concerns	Judgment comment: The method of measuring the outcome was appropriate and similar for the intervention and the control group. However, participants were aware of their received intervention, which could have influenced participant-reported outcomes.
Selection of the reported result	Some concerns	Judgment comment: The trial protocol was not available.
Overall	High risk of bias	

Study: O´Mahen et al, 2014 [33]

Bias	Authors' judgment	Support for judgment
Randomization process	Low risk of bias	Quote: 'Eligible and consenting women were randomized to receive either NetmumsHWD or TAU, minimized on depression severity (EPDS 513) and whether or not they were currently receiving pharmacological treatment. The minimization algorithm included a stochastic element to inform the allocation process and was administered remotely using a computer-generated code to ensure concealment. Randomization occurred online; eligible women were sent an electronic link to a webpage where they could learn their randomization assignment.'
Deviations from intended interventions	Low risk of bias	Judgment comment: Due to the nature of this intervention, blinding was not possible. No deviations from the intended intervention were reported. An appropriate analysis was used.
Missing outcome data	Some concerns	Quote: 'Post-treatment EPDS was completed by 37/41 (90%) women in the NetmumsHWD condition and by 34/42 (81%) women in the TAU group (χ^2 =1.45, p=0.23). A 6-month follow-up EPDS was completed by 31/41 (76%) women in the NetmumsHWD group and 28/41 (68%) women in the TAU group (χ^2 =0.37, p=0.47).'<br><br>Judgment comment: The loss to post-assessment could be related to participants' health status.
Measurement of the outcome	Some concerns	Judgment comment: The method of measuring the outcome was appropriate and similar for the intervention and control groups. However, participants were aware of their received intervention, which could have influenced participant-reported outcomes.
Selection of the reported result	Some concerns	Judgment comment: The trial protocol is not available, so it could not be checked whether results were analyzed according to a pre-specified analysis plan. A standardized questionnaire was used.
Overall	Some concerns	

Study: Stiles-Shields et al, 2019 [35]

Bias	Authors' judgment	Support for judgment
Randomization process	Low risk of bias	Quote: 'Randomization was created using PROC PLAN that randomly assigned participants in randomization blocks of six to either Boost Me (n = 10), Thought Challenger (n = 10), or waitlist control (n = 10). The randomized block design was used to ensure equal numbers were randomized to each group at a given time, should the study end early, or if there were seasonal effects. Once generated, this list was uploaded to Research Electronic Data Capture (REDCap), where study personnel were blinded to allocation prior to randomization, and participants would be randomized once eligibility was determined.'
Deviations from intended interventions	Some concerns	Quote: 'All Boost Me participants received the intervention. Three Thought Challenger participants did not receive the intervention; one reported not having enough device memory to download the app, and two were unresponsive to contact following randomization.'<br><br>Judgment comment: Due to the nature of this study, blinding was not possible.
Missing outcome data	Low risk of bias	Judgment comment: Nearly all outcome data of randomized participants are available. All Boost Me participants received the intervention. Three Thought Challenger participants did not receive the intervention; one reported not having enough device memory to download the app, and two were unresponsive to contact following randomization.
Measurement of the outcome	Some concerns	Judgment comment: A standardized questionnaire was used to assess the outcome. However, there were two active groups and one waitlist control group. Receiving an intervention probably had a different effect on the outcome than not receiving an intervention.
Selection of the reported result	Some concerns	There was no trial protocol, so it could not be checked whether results were analyzed according to a pre-specified analysis plan.
Overall	Some concerns	
