# Supplementary figures and images for: Internet-Based Behavioral Activation for Depression: Systematic Review and Meta-Analysis
Source: J Med Internet Res. 2023 May 25;25:e41643. doi: 10.2196/41643 (PMC10251223; doi:10.2196/41643)

## Multimedia Appendix 10: Funnel plot of the main analysis

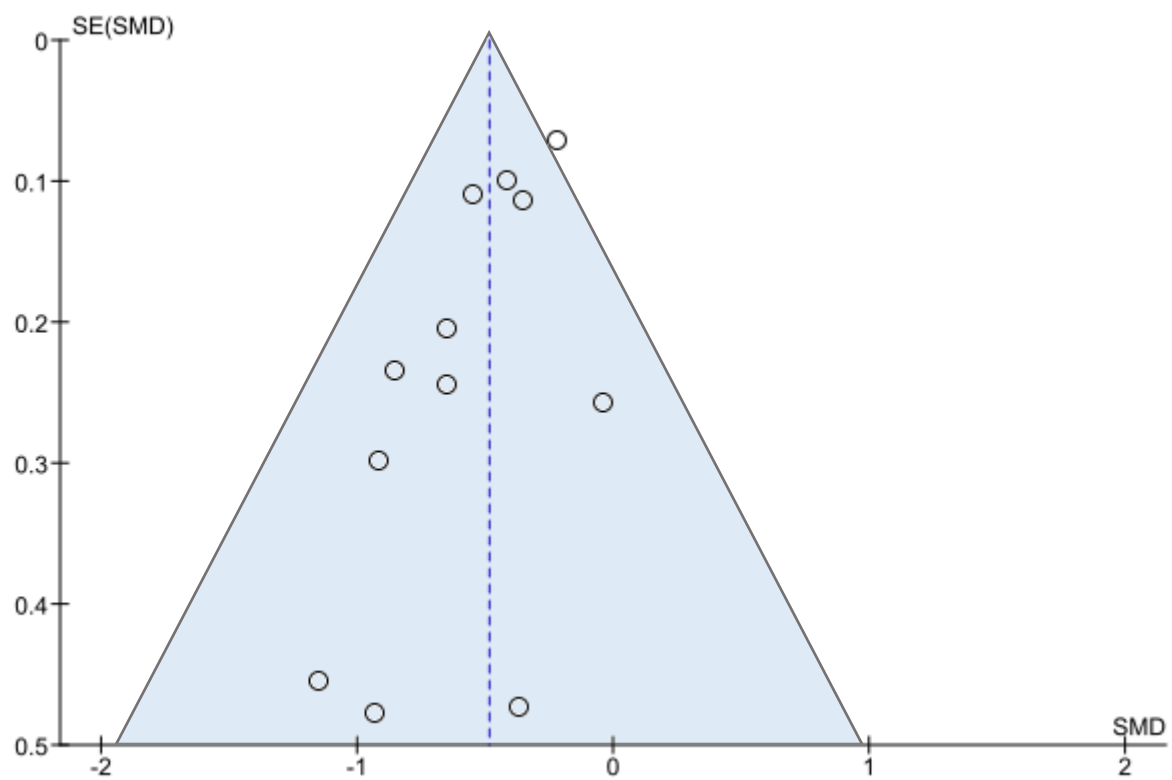

Supplement: Multimedia Appendix 10 [file jmir_v25i1e41643_app10.pdf]

## Multimedia Appendix 11. Forest plot of 6-months follow-up

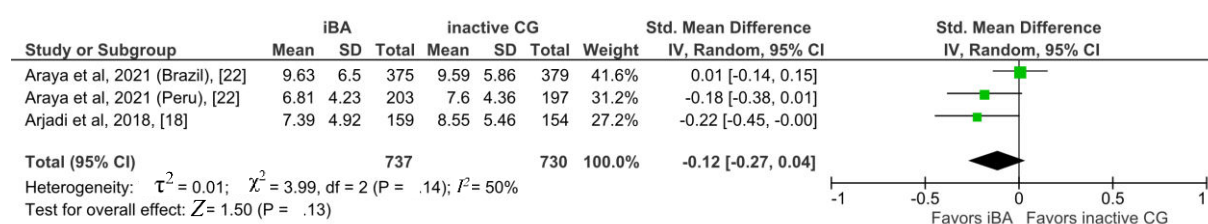

Supplement: Multimedia Appendix 11 [file jmir_v25i1e41643_app11.pdf]

## Multimedia Appendix 12. Forest plots of sensitivity analyses

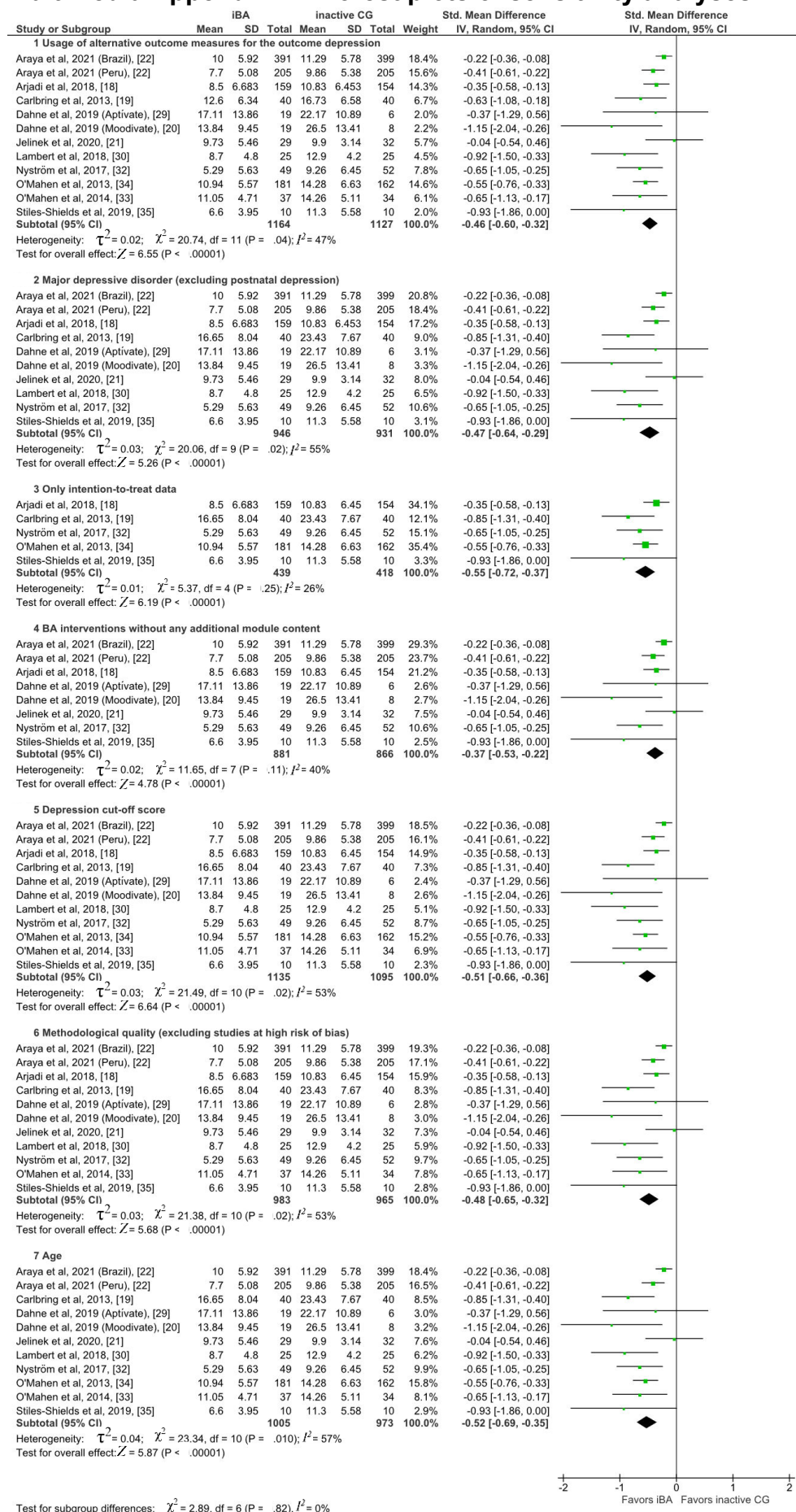

Supplement: Multimedia Appendix 12 [file jmir_v25i1e41643_app12.pdf]
