# Supplementary material for: Internet-Based Behavioral Activation for Depression: Systematic Review and Meta-Analysis
Source: J Med Internet Res. 2023 May 25;25:e41643. doi: 10.2196/41643 (PMC10251223; doi:10.2196/41643)

Multimedia Appendix 13. Forest plots for secondary outcomes anxiety, quality of life, and behavioral activation

A Effects if iBA in comparison to inactive control groups on anxiety

	iBA			inactive CG				Std. Mean Difference
Study or Subgroup	Mean	SD	Total	Mean	SD	Total	Weight	IV, Random, 95% CI
Carlbring et al, 2013, [19]	10.95	6.23	40	13.85	6.62	40	28.3%	-0.45 [-0.89, -0.00]
Lambert et al, 2018, [30]	7.1	3.8	25	10.9	3.7	25	16.0%	-1.00 [-1.59, -0.41]
Nyström et al, 2017, [32]	3.93	3.88	49	6.61	5.31	52	35.1%	-0.57 [-0.97, -0.17]
O'Mahen et al, 2014, [33]	8.71	4.61	31	11.29	5.49	28	20.6%	-0.50 [-1.02, 0.02]
**Total (95% CI)**			**145**			**145**	**100.0%**	**-0.59 [-0.83, -0.35]**

Heterogeneity: τ^2 = 0.00; χ^2 = 2.34, df = 3 (P = .50); I^2 = 0%

Test for overall effect: Z = 4.90 (P < .00001)

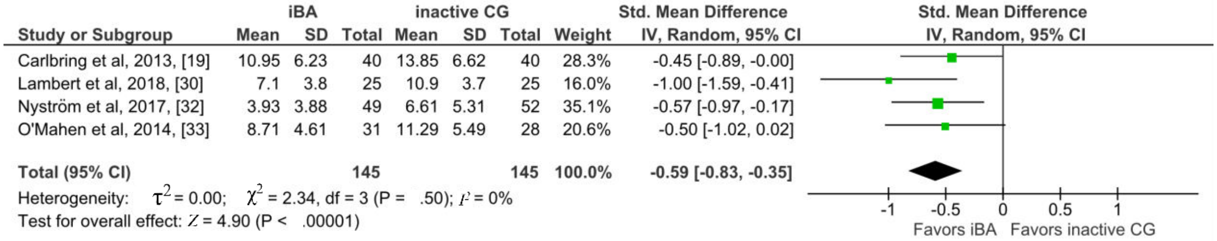

B Effects of iBA in comparison to inactive control groups on quality of life

	iBA			inactive CG				Std. Mean Difference
Study or Subgroup	Mean	SD	Total	Mean	SD	Total	Weight	IV, Random, 95% CI
Araya et al, 2021 (Brazil), [22]	0.68	0.19	389	0.65	0.19	396	47.7%	0.16 [0.02, 0.30]
Araya et al, 2021 (Peru), [22]	0.7	0.2	209	0.65	0.24	203	24.9%	0.23 [0.03, 0.42]
Arjadi et al, 2018, [18]	79.31	14.12	159	75.39	12.04	154	18.8%	0.30 [0.07, 0.52]
Carlbring et al, 2013, [19]	0.78	1.57	40	0.75	1.77	40	4.9%	0.02 [-0.42, 0.46]
Jelinek et al, 2020, [21]	3.28	0.88	29	3.12	0.66	32	3.7%	0.20 [-0.30, 0.71]
**Total (95% CI)**			**826**			**825**	**100.0%**	**0.20 [0.10, 0.29]**

Heterogeneity: τ^2 = 0.00; χ^2 = 1.82, df = 4 (P = .77); I^2 = 0%

Test for overall effect: Z = 3.97 (P < .0001)

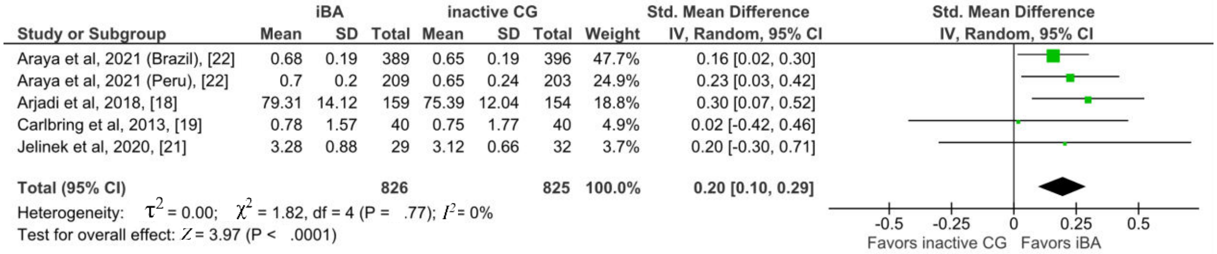

C Effects of iBA in comparison to inactive control groups on behavioral activation

	iBA			inactive CG				Std. Mean Difference
Study or Subgroup	Mean	SD	Total	Mean	SD	Total	Weight	IV, Random, 95% CI
Araya et al, 2021 (Brazil), [22]	25.97	8.4	388	25.14	8.79	383	45.5%	0.10 [-0.04, 0.24]
Araya et al, 2021 (Peru), [22]	29.52	9.14	208	25.95	9.13	205	39.4%	0.39 [0.20, 0.58]
Jelinek et al, 2020, [21]	85.76	22.9	29	78.59	21.63	32	15.1%	0.32 [-0.19, 0.82]
**Total (95% CI)**			**625**			**620**	**100.0%**	**0.25 [0.01, 0.48]**

Heterogeneity: τ^2 = 0.03; χ^2 = 5.93, df = 2 (P = .05); I^2= 66%

Test for overall effect: Z = 2.08 (P = .04)

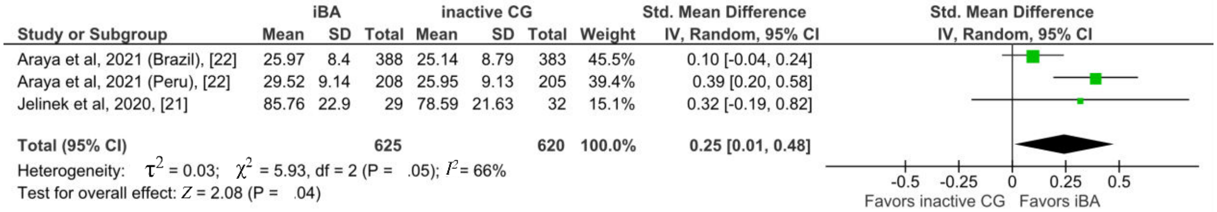

Supplement: Multimedia Appendix 13 [file jmir_v25i1e41643_app13.pdf]
